# Diet and Maternal Obesity Are Associated with Increased Oxidative Stress in Newborns: A Cross-Sectional Study

**DOI:** 10.3390/nu14040746

**Published:** 2022-02-10

**Authors:** Arturo Lopez-Yañez Blanco, Keyla M Díaz-López, Jenny Vilchis-Gil, Hector Diaz-Garcia, Jacqueline Gomez-Lopez, Patricia Medina-Bravo, Javier T Granados-Riveron, Juan M Gallardo, Miguel Klünder-Klünder, Rocío Sánchez-Urbina

**Affiliations:** 1 Unidad de Investigación en Malformaciones Congénitas, Hospital Infantil de México Federico Gómez, Mexico City 06720, Mexico; arturolyb@gmail.com (A.L.-Y.B.); keyla_yinyer@hotmail.com (K.M.D.-L.); hecdiazgar@gmail.com (H.D.-G.); javiertgranados@gmail.com (J.T.G.-R.); 2 Unidad de Investigación Epidemiológica en Endocrinología y Nutrición, Hospital Infantil de México Federico Gómez, Mexico City 06720, Mexico; jvilchisgil@gmail.com; 3 Facultad de Medicina, Universidad Nacional Autónoma de México, Mexico City 04510, Mexico; 4 Hospital Militar de Especialidades de la Mujer y Neonatología, Secretaria de la Defensa Nacional, Mexico City 11200, Mexico; drajackiegomezlopez@yahoo.com.mx; 5 Departamento de Endocrinología, Hospital Infantil de México Federico Gómez, Mexico City 06720, Mexico; drapatty_75@hotmail.com; 6 Unidad de Investigación Medica en Enfermedades Nefrológicas, Hospital de Especialidades Centro Médico Nacional Siglo XXI, Instituto Mexicano del Seguro Social, Mexico City 06720, Mexico; jmgallardom@gmail.com; 7 Subdirección de la Gestión de la Investigación, Hospital Infantil de México Federico Gómez, Mexico City 06720, Mexico; klunderk@gmail.com; 8 Escuela Superior de Medicina, Instituto Politécnico Nacional, Mexico City 11340, Mexico

**Keywords:** maternal obesity, oxidative stress, dietary intake, Malondialdehyde, Nitric Oxide

## Abstract

Overweight and obesity have become a world-health public problem, mainly for developing countries. Both health conditions have a higher prevalence among women of childbearing age. Physiopathology, overweight and obesity are characterized by a chronic oxidative stress status, which has deleterious effects on mothers and children. Hence, we determine whether the qualities of diet during pregnancy and maternal pregestational body mass index (BMI) are associated with increased oxidative stress markers in mothers and newborns. Two hundred forty-two (242) mother-newborn pairs were classified according to their pregestational BMI. Information on food intake was collected using a food frequency questionnaire in the third trimester of pregnancy. Levels of Malondialdehyde (MDA) and Nitric Oxide (NO) were measured in plasma from mothers at the end of the third trimester of pregnancy and from cord blood at birth. MDA and NO levels in mother–newborn pairs with maternal pregestational overweight or obesity were higher than in mother–newborn pairs with pregestational normal weight. For women (and newborns) who had a higher intake of fruit and vegetables, the levels of NO and MDA were lower. Lastly, women with pregestational obesity had lower fruit and vegetable intake during pregnancy and higher levels of oxidative stress and in their newborns.

## 1. Introduction

Overweight and obesity result from a persistent increase in a positive energy balance associated with a modern sedentary lifestyle and nutrition richer in quantity but lower in quality. Obesity in adults is a risk factor for metabolic diseases, cardiovascular diseases, type 2 diabetes and other chronic diseases [1]. During pregnancy, obesity rates vary among ethnic groups, but obesity is present in a large population of Hispanic women [2]. In Mexico, for women of childbearing age (aged 12 to 49), the prevalence of obesity reaches up to 47.6% [3]. Obesity during pregnancy has been associated with an increased risk of metabolic syndrome later in postnatal life during adolescence [4]. In addition, obesity during pregnancy increases the risk of metabolic syndrome in subsequent pregnancies [5].

Maternal obesity predisposes newborns to a state of oxidative stress (OS) [6], which is detrimental to cellular functions and has potential mutagenic and teratogenic effects [7]. Increased OS based on the measurement of specific metabolic markers such as Malondialdehyde (MDA) and Nitric Oxide (NO) has been observed in subjects with obesity [8,9]. MDA is largely derived from the peroxidation of polyunsaturated fatty acids and serves as a tissue damage marker with the capacity to induce mutations and DNA damage [10]. Moreover, NO has antioxidant as well as pro-oxidant actions in cells. Endothelial NO is a potent vasodilator agent with antihypertensive, antithrombotic, antiatherogenic and antiproliferative properties in smooth muscle. However, high levels of endothelial NO and/or peroxynitrite are associated with proinflammatory responses and tissue damage [9,11].

Nevertheless, OS is necessary for placental differentiation and embryonic development in early gestation, and it promotes cellular proliferation and differentiation. However, excess OS is associated with placental, embryonic and foetal pathologies [12]. Therefore, maternal obesity during gestation has been proposed to be detrimental to the foetus during the early stages of development, through teratogenesis induced by increasing OS [13]. Although the aetiology of teratogenic mechanisms in newborns has not been completely clarified to date, OS effects may be associated. In addition, for women with pregestational obesity, a nutrition restriction with reduced nutrient intake has been associated with potential damage to embryonic development [7]. 

In addition, the deficient consumption of certain micronutrients and maternal obesity may modify the duration of gestation and foetal development and growth, which may be associated with pregnancy loss, premature birth, delay in intrauterine growth, congenital anomalies and long-term metabolic alterations, such as insulin resistance, type 2 diabetes and cardiovascular diseases [14]. Interestingly, studies in mouse models of obesity and pregnant mice have shown that food supplementation with antioxidants (vitamin E, dimethylglycine and vitamin B complex) decreases OS markers [15,16,17], whereas micronutrient deficiencies of vitamin B12 in pregnant mice increase adiposity and lipid peroxidation [18].

Due to the limited information on the effects of pregestational nutritional status on OS markers in women and their offspring, the present study aimed to determine the potential association of pregestational BMI and diet quality during pregnancy with OS marker levels of mothers at end pregnancy and their offspring at delivery.

## 2. Materials and Methods

### 2.1. Subjects

A total of 242 healthy pregnant women were consecutively recruited during the third trimester of pregnancy for this cross-sectional study, which was conducted between January 2015 and July 2016. All pregnant women signed written informed consent. This study was conducted in accordance with the latest version of the principles of the Declaration of Helsinki as well as the relevant Mexican legislation. The inclusion criteria were as follows: women who delivered with a gestational age of 38–40 weeks from the last menstruation and had adequate prenatal control, including monthly medical consultation starting in the first trimester of pregnancy and with a minimum of two normal prenatal ultrasounds. Women who had pre-eclampsia; diabetes mellitus type 1 or 2; or gestational diabetes mellitus were excluded. Data on the weight of women before and at the end of pregnancy were collected during medical examination, and a physician measured the height of women with a stadiometer (SECA^®^, Hamburg, Germany) at the time of recruitment; likewise, perinatal history was collected based on direct questions to patients during medical examination. Anthropometric data on weight and length, weeks of gestation, natural delivery or caesarean section, sex and Apgar score of the newborns with the perinatal medical history were collected (Table 1). The mother–newborn pairs were classified according to the pregestational body mass index (BMI) with normal weight (BMI: 18.5 to 24.9 kg/m^2^, *n* = 173); with overweight (BMI: 25.0 to 29.9 kg/m^2^, *n* = 74); or with obesity (BMI: ≥30 kg/m^2^, *n* = 35).

### 2.2. Food Frequency Questionnaire

At the time of patient recruitment (third trimester of pregnancy), a food frequency questionnaire (FFQ) to assess regular food intake during the pregnancy was administered to all participants by the medical staff. The questionnaire was validated for estimated folate intake in the Mexican population [20]. The questionnaire contained 127 food items. As support material, the interviewer used food replicas in order to standardize the types and amounts of the main food groups consumed by the participants. Participants’ food intake per day was estimated, and the amount of food consumed was calculated in terms of units of measurement (e.g., piece, cup, plate or spoon) and the size of the unit (e.g., small, medium or large). For the analysis, the frequencies of consumption were calculated in grams or milliliters ingested per day for each of 127 food items. The participants’ daily intake levels of energy, macronutrients and micronutrients were calculated using the Food Processor SQL program (version 10.9.0, 2011) and Mexican food tables including data on traditional Mexican food [21]. The percentages of adequate intake of energy, macronutrients and micronutrients were calculated by using the recommended Dietary Reference Intakes (DRI) [22]. For the analysis, data cleaning was carried out, and the consumptions of energy, macronutrients and micronutrients greater than 5 standard deviations of the average consumption were not considered plausible. Concerning lower values, energy adequacy values less than 25% were eliminated, as adequacy percentages less than that could not represent an intake compatible with life.

### 2.3. Plasma Samples

In the last medical examination just before delivery, 5 mL peripheral blood samples were collected. Immediately after birth, the umbilical cords were clamped and 5 mL blood samples were collected. The samples were collected in ethylenediaminetetraacetic acid-containing tubes and centrifuged at 1300× *g* for 15 min at 4 °C to separate the plasma, which was stored at −20 °C until biochemical analyses were performed. 

### 2.4. Nitric Oxide (NO)

NO was measured by determining the total quantity of nitrite (NO^2−^), which is the stable product of NO metabolism in plasma. The Griess reagent, an aqueous solution of 1% sulfanilamide (Sigma-Aldrich^®^, Darmstadt, Germany) and 0.1% naphthylethylenediamine (Sigma-Aldrich^®^, Darmstadt, Germany) in 2.5% H3PO4 (JT Baker^®^, Philadelphia, PA, USA), forms a stable chromophore with NO^2−^. The reaction’s absorbance was determined at 546 nm in a xMark Microplate Absorbance Spectrophotometer (BIORAD, Berkeley, CA, USA). The calibration curve was constructed using different concentrations of sodium nitrite dissolved in 0.9% NaCl.

The tertiles of NO were constructed based on concentrations in mothers and newborns. The tertiles of NO for the mothers were as follows: from 1.0 to 6.5 µmol/L, tertile 1; from 6.8 to 22.8 µmol/L, tertile 2; and from 23.0 to 384.4 µmol/L, tertile 3. The tertiles of NO for the newborns were as follows: from 0 to 9.6 µmol/L, tertile 1; from 10.2 to 29.1 µmol/L, tertile 2; and from 29.2 to 231.9 µmol/L, tertile 3.

### 2.5. Malondialdehyde (MDA)

Lipid peroxidation was assessed by measuring MDA levels in samples via the thiobarbituric acid (TBA)-reactive substances test and was calculated as millimoles per liter (mmol/L). Briefly, 200 mL 25% trichloroacetic acid (JT Baker^®^, Philadelphia, PA, USA) was added to an aliquot of plasma of 100 μL. The samples were incubated at 48 °C for 15 min, followed by centrifugation at 2800× *g* for 3 min. The supernatants (100 mL) were neutralized with 1 mL of 4 M NaOH (JT Baker^®^, Philadelphia, PA, USA) and 1 mL 0.7% TBA (Acros Organics, Geel, Belgium). The mixture was incubated at 90 °C for 60 min. The absorbance of the color reaction in the organic phase (1-butanol, Sigma-Aldrich^®^, St. Louis, MO, USA) was measured at a wavelength of 532 nm using a xMark Microplate Absorbance Spectrophotometer (BIORAD, Berkeley, CA, USA). Tetramethoxypropane was used as the standard.

The tertiles of MDA were constructed based on the concentrations in mothers and newborns. The tertiles of MDA for the mothers were as follows: from 0 to 11.8 µmol/L, tertile 1; from 11.9 to 30.3 µmol/L, tertile 2; and from 30.8 to 707.9 µmol/L, tertile 3. The tertiles of MDA for the newborns were as follows: from 0 to 0.72 µmol/L, tertile 1; from 0.74 to 26.1 µmol/L, tercile 2; and from 28.4 to 700.8 µmol/L, tertile 3.

### 2.6. Statistical Analysis

Descriptive statistics, including means, standard deviation and frequencies, were used to describe the baseline characteristics of mothers and newborns. An exploratory analysis of all variables was carried out in order to identify their distribution. Continuous quantitative variables that did not have a normal distribution were analysed using non-parametric methods. The sample size for the variables NO and MDA was estimated using the ratio comparison formula (α = 0.05, β = 0.90) based on the data by Gallardo et al. (2015) [6]. Analysis of variance test for continuous variables and Pearson’s χ^2^ test for categorical data were used to compare differences between groups. Median values with 25th and 75th percentiles were determined to describe the intake of energy, macronutrients, micronutrients, fruits and vegetables, and the Kruskal–Wallis test was used for comparisons among groups. The Kruskal–Wallis test was also used to evaluate OS markers in mother–newborn pairs based on the pregestational nutritional diagnosis of the mother. Pearson’s correlation test was used to assess the correlation of NO and MDA concentrations in mother-newborn pairs. The Kruskal–Wallis and χ^2^ tests were used to compare the intake of fruits, vegetables and vitamins and folic acid supplementation among the tertiles of OS markers after the data were adjusted by calorie intake and maternal BMI before pregnancy. All statistical analyses were performed using the STATA/SE 11.0 statistical programme (STATA Corp., College Station, TX, USA). *p*-values lower than 0.05 were considered to indicate statistically significant differences. 

## 3. Results

### 3.1. Clinical Characteristics of Mothers and Newborns

The perinatal characteristics of mothers and the characteristics of the newborns at birth were categorized according to the pregestational maternal BMI (Table 1). The mean age of women with normal weight was significantly different than those of women with overweight and obesity (*p* = 0.002). Weight gain during pregnancy was lower in women with obesity compared to those with normal weight (*p* = 0.002). In contrast, the newborns of obese women were taller than those of women with normal weight (*p* = 0.015), even though the mean weight of newborns was not different between the two groups.

More than 90% of the women indicated that they consumed folic acid supplements in the perinatal period; the rate of supplementation was not significantly different among the groups (*p* = 0.34). However, significantly more women with normal weight (39.4%) consumed folic acid supplements in the first four weeks of pregnancy compared to those with overweight or obesity (21.4%, and 14.4%, respectively; *p* = 0.016) (Table 1).

### 3.2. Micronutrients and Macronutrients Consumption

The evaluation of nutritional status revealed that the women with normal weight had higher calorie intake from macronutrients and micronutrients as well as higher folic acid intake (*p* < 0.05) and vegetables (*p* < 0.05) compared to those with obesity (Table 2).

### 3.3. Levels of Oxidative Stress (MDA and NO)

The analysis of OS markers revealed differences among the three groups. The levels of NO were lower in mothers with normal weight than in those with overweight and obesity (*p* < 0.001). Additionally, the levels of MDA were higher in women with obesity compared to those with normal weight and those that are overweight. Among newborns, the levels of NO and MDA were higher in those born to mothers with obesity compared to those born to mothers with normal weight and those with overweight (Table 3).

Positive correlations between mother age and newborns MDA (R^2^ = 0.151, *p* = 0.03) and mother age with mother NO (R^2^ = 0.213, *p* = 0.01) were found in this study. 

**Figure 1:**
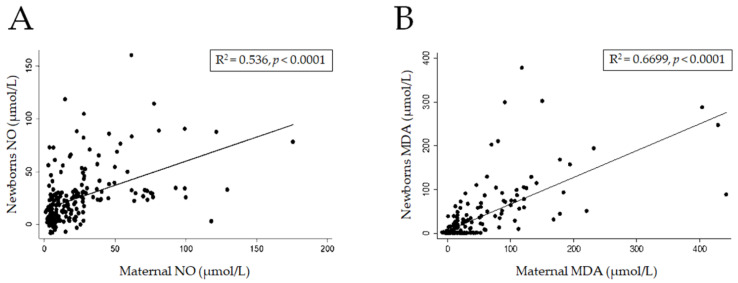
Correlation of stress oxidative between mothers and newborn binomials. Correlation of Nitric Oxide (**A**) and Malondialdehyde (**B**) levels.

A positive correlation in NO levels was found between the newborns and their mothers (R^2^ = 0.54; *p* < 0.001). Similarly, a correlation in MDA levels was observed between newborns and mothers (R^2^ = 0.67; *p* < 0.001) (Figure 1A,B).

### 3.4. Fruit, Vegetables and Vitamins Consumption

The intakes of fruits, vegetables, vitamins and folic acid supplementation (FAS) of mothers according to NO and MDA tertiles of mothers and newborns are summarized in Table 4.

We observed that mothers in tertiles 2 and 3 of NO and MDA had a lower intake of fruits, vegetables and vitamins than those in tertile 1. Consistent with previously described results, the mother’s newborns in tertiles 2 and 3 of NO and MDA had a lower intake of fruits, vegetables and vitamins in contrast with the mother’s newborns in tertile 1.

We observed that most of the women who received FAS during the first fourth weeks of pregnancy had NO and MDA levels in tertile 1 (47.8% and 44.8%, respectively), while only 7.4% and 13.4% of them were in tertile 3, respectively. In contrast, for mothers who received FAS after the 20th week of pregnancy, the percentages of the NO and MDA levels in tertile 3 was higher (56.6% and 59.1%, respectively) in comparison to those in tertiles 1 or 2. Accordingly, most of the newborns of mothers who received FAS before the fourth week had NO and MDA levels in tertile 1 (56.9% and 50.0%, respectively), and most of the newborns of mothers who received FAS after the 20th week had NO and MDA levels in tertile 3 (47.8% and 43.1%, respectively).

The previously mentioned differences were independent of the total caloric intake and prepregnancy BMI.

## 4. Discussion

During normal pregnancy, pro-oxidant and antioxidant molecules are produced, and their balance promotes development and physiological functions in the embryo and in the foetus [12]. NO is a marker of OS, and during pregnancy, the synthesis of NO is regulated by the placenta, and its levels increase in the later stages of normal pregnancy [23]. MDA is a product of lipoperoxidation and a marker of OS, and it interacts with various components, such as DNA, resulting in deleterious effects on biological functions [10]. In the present study, we observed that the plasma levels of NO and MDA were higher in mothers with pregestational overweight or obesity, which correlated with the respective OS marker levels in their offspring (Figure 1A,B). Those findings suggested that there is an association with the increase in levels of mother–child pairs with regard to BMI in women with overweight and obesity (*p* < 0.01) (Table 3), which is an effect only previously described in women with pregestational obesity [6,24,25]. This was an important finding because gestation itself is a state of elevated OS, which is strictly regulated by several mechanisms to minimize the damage that may be caused by OS. However, increases in OS markers are related to cellular ageing, DNA damage, teratogenic effects and mutagenic effects during embryonic and foetal development [26]. These findings may be associated with epigenetic changes in the offspring of women with obesity, because OS may alter gene expression during cellular differentiation, and the effect may be sustained, starting in the prenatal period and continuing throughout life [27]. 

Maternal obesity and high-fat diets are associated with maternal metabolic conditions, including increased levels of OS markers, leptin, insulin, glucose, triglycerides and inflammatory cytokines [24], which are associated with excess adiposity during pregnancy that affects foetal programming. Several epidemiological studies have shown that exposure to a suboptimal nutritional environment (excessive maternal calorie intake or poor micronutrient intake) and increased levels of OS during early life stages are associated with an increased risk for obesity and other comorbidities, such as type 2 diabetes, insulin resistance and cardiovascular diseases [28]. In fact, current evidence supports the hypothesis that obesity in adult life begins in early stages of development and has inter and transgenerational effects.

We observed that pregnant mothers with obesity modify nutritional habits by reducing food intake and thus fruit and vegetable intake (Table 2), which are sources of vitamins that may have an antioxidant effect [29], which is in accordance with previous reports [7]. Furthermore, it is important to mention that we did not observe an association among maternal BMI, OS levels and fruit and vegetable intake (*p* > 0.05). Nevertheless, analyses of the entire study group indicated that higher intakes of fruits and vegetables resulted in lower levels of NO and MDA (Table 4), which is consistent with previous reports, showing that a higher intake of vitamin A, B12, C, E and folic acid decreases the plasma levels of NO and MDA in adults [30].

In the present study, maternal intake of folic acid after 20 weeks of gestation was associated with increased NO levels in newborns and increased maternal MDA levels compared to the mothers who received folic acid supplementation during the first four weeks of gestation (Table 4). This finding may be related to the multiple roles folic acid plays in OS, including its direct antioxidant effect through the regulation of NO levels [31] and through decreasing MDA formation [32]. Importantly, decreased levels of OS markers have been observed when folic acid supplements are given at doses of 0.4 to 10 mg to adult populations [33]. Similarly, the present study showed that lower plasma levels of NO and MDA were present in pregnant women who received folic acid supplementation during the first 20 weeks of pregnancy (Table 4) [19]. These findings may be associated with adherence to treatment with folic acid [34]. However, the present study only revealed associations and did not elucidate direct effect of folic acid on the regulation of NO and MDA.

The global increases in maternal obesity and Western diets rich in saturated fats may potentially increase neurodevelopmental and neuropsychological morbidities in the next generation: Epidemiological studies have provided evidence of associations between maternal obesity and adverse neurological and psychiatric developmental outcomes in offspring [35].

Medical care related to weight gain and guidelines on nutrition, micronutrient and macronutrient supplementation for women during pregnancy are based on global guidelines proposed by the World Health Organization, United Nations Children’s Fund and the US Institute of Medicine. These standards comprising recommendations to reduce disease risk for mothers and children should be followed even more strictly for women with pregestational overweight or obesity and for those with poor intake of micronutrients. Timely medical care should be provided to advise women to have a healthier diet during pregnancy, including increased intakes of fruits, vegetables, whole grains, low-fat dairy products and a variety of protein sources before pregnancy [36]. Health providers should encourage patients to consume healthy food, and empty calories should be minimized or discouraged. These diet recommendations should aid women meeting their nutritional needs without exceeding their calorie requirements [37]. The mothers’ energy intake values were estimated by using FFQ, and mothers with normal prepregnancy weight had higher energy intake than mothers with overweight or with obesity. Similar findings have been reported in a nutritional national survey in which FFQ was used [38]; in this prior survey, individuals with obesity reportedly ingested fewer calories, a finding that was considered to be underreported. Therefore, the explanation of this paradox may be an underreporting of food consumption. 

The main limitation of the present study was that maternal pregestational BMI (pre-BMI) could not be established as the cause of OS levels in newborns due to the cross-sectional design of the study. Moreover, due to the genetic [39] and epigenetic [40] effects on redox homeostasis, it is complicated to attribute the decreasing OS effect to fruit or vegetable consumption or folic acid supplementation. However, the present study clearly showed positive correlations of oxidative stress in mother–newborn pairs.

## 5. Conclusions

The pre-BMI of mothers had a positive effect on oxidative stress markers (NO or MDA) of the mother–newborn pairs at the end of pregnancy. Furthermore, oxidative stress markers were correlated between mothers and newborns. In contrast, low consumption of fruit and vegetables or a delay in folic acid supplementation during pregnancy, independent of pre-BMI or caloric consumption in pregnancy, caused increases in oxidative stress in mother–newborn pairs.

## Figures and Tables

**Table 1:** Women and newborns clinic characteristics according to the pregestational BMI.

Mothers	Normal Weight Mean ± SD (*n* = 133)	Overweight Mean ± SD (*n* = 74)	Obesity Mean ± SD (*n* = 35)	*p*
Age (y)	23.2 ± 5.2	25.9 ± 5.9	25.5 ± 5.7	0.002 ^†^
Height (cm)	157.1 ± 5.6	156.1 ± 5.8	155.6 ± 7.1	0.377 ^†^
Weight before pregnancy (kg)	54.5 ± 8.0	66.3 ± 6.0	77.1 ± 8.4	<0.001 ^†^
BMI (kg/m^2^)	22.0 ± 2.0	27.2 ± 1.3	31.8 ± 2.0	<0.001 ^†^
Weight gain (kg)	11.7 ± 4.8	10.7 ± 5.9	7.9 ± 5.1	0.002 ^†^
Perinatal folic acid supplementation ^&^, n (%)	127 (97.0)	70 (94.6)	32 (91.4)	0.340 ^‡^
0–4 weeks of gestation, n (%) 5–20 weeks of gestation, n (%) >20 weeks of gestation, n (%)	50 (39.4) 67 (52.8) 10 (7.9)	15 (21.4) 46 (65.7) 9 (12.9)	5 (14.3) 25 (71.4) 5 (14.3)	0.016 ^‡^
**Newborns**				
Weight (kg)	3.1 ± 0.4	3.2 ± 0.4	3.2 ± 0.5	0.159 ^†^
Length (cm)	48.9 ± 2.0	49.5 ± 1.8	49.7 ± 2.0	0.015 ^†^
Weeks of gestation	38.8 ± 1.5	39.0 ± 1.2	38.4 ± 1.3	0.153 ^†^
Type of birth/caesarean, n (%)	32 (26.5)	29 (40.3)	12 (35.3)	0.126 ^‡^
Apgar score at 1 min (≤ 8)	5 (3.8)	6 (8.1)	5 (14.3)	0.069 ^‡^
Sex, male, n (%)	62 (48.1)	34 (46.0)	19 (54.3)	0.716 ^‡^

^†^ Analysis of variance test and ^‡^ Pearson’s χ^2^ test. ^&^ 0.4–4.0 mg/day [19]. BMI, Body Mass Index; SD, standard deviation.

**Table 2:** Women intake of energy, nutrients and food groups during pregnancy.

Daily Nutrient Intake	Normal Weight Median (p25, p75) (n = 131)	Overweight Median (p25, p75) (n = 73)	Obesity Median (p25, p75) (n = 34)	*p* ^†^
Energy, (kcal/d)	2282 (1737, 3053)	1793 (1590, 2393)	1711 (1514, 2420)	0.002
Protein, (g/d) % kcal/d	101 (68, 139) 17.0 (15.4, 18.3)	76 (61, 113) 15.7 (14.5, 18.1)	74 (58, 99) 17.0 (13.8, 20.3)	0.005 0.275
Carbohydrates, (g/d) % kcal/d	325 (257, 456) 56.2 (51.9, 59.6)	284 (246, 369) 59.0 (54.3, 61.1)	276 (217, 374) 59.9 (56.3, 64.0)	0.045 0.001
Lipids, (g/d) % kcal/d	72 (53, 96) 27.2 (24.3, 29.8)	55 (45, 75) 25.1 (23.1, 27.3)	49 (40, 63) 23.5 (21.3, 25.8)	<0.001 <0.001
Folate (μg/d) % adequacy ^‡^	255 (184, 379) 79.8 (57.6, 118.5)	199 (169, 267) 62.3 (52.7, 83.5)	190 (153, 251) 59.4 (47.7, 78.4)	0.002 0.002
Vitamin B12 (μg/d) % adequacy ^‡^	4.1 (2.7, 7.4) 206 (134, 370)	3.1 (2.2, 5.1) 156 (109, 257)	2.5 (1.9, 5.4) 127 (96, 271)	0.005 0.005
Vitamin A (RE/d) % adequacy ^‡^	799 (545, 1129) 160 (109, 226)	578 (453, 982) 116 (91, 196)	511 (370, 840) 102 (74, 168)	<0.001 <0.001
Vitamin C (mg/d) % adequacy ^‡^	171 (119, 238) 258 (198, 397)	163 (130, 201) 270 (215, 334)	154 (125, 204) 257 (207, 340)	0.540 0.540
Vitamin E (mg/d) % adequacy ^‡^	4.5 (3.4, 5.5) 37 (28, 46)	4.0 (3.4, 4.9) 34 (28, 41)	3.8 (2.9, 4.8) 32 (24, 40)	0.069 0.069
Consumption of fruits and vegetables				
Fruit (g/d)	288 (173, 445)	272 (197, 395)	258 (175, 399)	0.557
Vegetables (g/d)	229 (158, 313)	185 (140, 254)	157 (116, 194)	0.004

^†^ Kruskal–Wallis test. ^‡^ Recommendations of Dietary Reference Intakes [22]. *p*, percentile; RE, retinol equivalents.

**Table 3:** Oxidative stress markers in mothers and newborns according to the pregestational BMI.

	**Maternal NO (** **µmol/L)** ^†^			***p***		**Maternal MDA (** **µmol/L)** ^†^			***p***
	**Normal weight**	**Overweight**	**Obesity**			**Normal weight**	**Overweight**	**Obesity**	
p25	5.1	6.3	15.0	<0.001	p25	5.3	3.7	8.4	<0.001
Median	7.9	17.4	27.9		Median	16.8	16.4	104.1	
p75	21.1	49.9	51.5		p75	29.4	47.9	129.1	
	**Newborn NO (** **µmol/L)** ^ † ^			***p***		**Newborn MDA (** **µmol/L)** ^ † ^			***p***
	**Normal weight**	**Overweight**	**Obesity**			**Normal weight**	**Overweight**	**Obesity**	
p25	5.5	9.6	15.9	0.001	p25	0.6	0.7	2.3	<0.001
Median	14.2	22.1	53.5		Median	0.8	15.3	73.3	
p75	30.0	30.9	84.3		p75	19.9	51.6	114.7	

^†^ Kruskal–Wallis test. *p*, percentile; BMI, Body Mass Index; NO, Nitric Oxide; MDA, Malondialdehyde.

**Table 4:** Intake of fruits, vegetables and vitamins according to tertiles of Nitric Oxide and Malodialdehyde levels in mothers and newborns.

	**Nitric Oxide (µmol/L) ^†^**					
	**Mothers**			**Newborns**		
	**Tertile 1** (1.0 to 6.5) p50 (p25, p75)	**Tertile 2** (6.8 to 22.8) p50 (p25, p75)	**Tertile 3** (23.0 to 384.4) p50 (p25, p75)	**Tertile 1** (0 to 9.6) p50 (p25, p75)	**Tertile 2** (10.2 to 29.1) p50 (p25, p75)	**Tertile 3** (29.2 to 231.9) p50 (p25, p75)
Fruits (g/d)	336 (272, 419)	293 (234, 369)	242 (215, 271)	328 (253, 398)	297 (234, 363)	252 (214, 310)
Vegetables (g/d)	235 (198, 299)	207 (162, 262)	166 (150, 193)	224 (183, 271)	211 (164, 262)	177 (150, 217)
Folate (μg/d)	296 (224, 391)	247 (178, 334)	189 (157, 221)	285 (201, 367)	253 (178, 328)	201 (157, 267)
Vitamin A (ER/d)	784 (635, 1045)	675 (493, 902)	508 (448, 615)	741 (572, 934)	699 (497, 886)	554 (448, 722)
Vitamin C (mg/d)	187 (153, 220)	167 (144, 202)	149 (135, 163)	184 (149, 210)	167 (147, 197)	155 (135, 179)
Vitamin B12 (μg/d)	4.2 (3.4, 5.7)	3.6 (2.6, 4.9)	2.6 (2.3, 3.2)	4.0 (3.0, 5.0)	3.7 (2.6, 4.8)	2.9 (2.3, 3.9)
Vitamin E (mg/d)	4.7 (3.9, 5.6)	4.2 (3.5, 5.0)	3.6 (3.3, 3.9)	4.6 (3.7, 5.4)	4.2 (3.5, 4.9)	3.8 (3.3, 4.3)
**Perinatal folic acid supplementation**						
0–4 weeks of gestation, n (%)	32 (47.8)	30 (44.8)	5 (7.4)	33 (56.9)	17 (29.3)	8 (13.8)
5–20 weeks of gestation, n (%)	32 (25.2)	39 (30.7)	56 (44.1)	25 (21.6)	44 (37.9)	47 (40.5)
>20 weeks of gestation, n (%)	4 (17.4)	6 (26.1)	13 (56.5)	5 (21.8)	7 (30.4)	11 (47.8)
	**Malondialdehyde (µmol/L) ^†^**					
	**Mothers**			**Newborns**		
	**Tertile 1** (0 to 11.8) p50 (p25, p75)	**Tertile 2** (11.9 to 30.3) p50 (p25, p75)	**Tertile 3** (30.8 to 707.9) p50 (p25, p75)	**Tertile 1** (0 to 0.72) p50 (p25, p75)	**Tertile 2** (0.74 to 26.1) p50 (p25, p75)	**Tertile 3** (28.4 to 700.8) p50 (p25, p75)
Fruits (g/d)	325 (246, 422)	398 (257, 366)	245 (209, 275)	339 (283, 419)	297 (237, 366)	247 (216, 276)
Vegetables (g/d)	217 (171, 294)	223 (186, 262)	172 (149, 193)	239 (198, 294)	211 (166, 263)	173 (149, 191)
Folate intake (μg/d)	283 (1934, 393)	277 (207, 332)	191 (152, 228)	300 (235, 391)	253 (183, 332)	192 (157, 229)
Vitamin A intake (REd)	733 (531, 1034)	731 (586, 898)	529 (432, 614)	815 (636, 1032)	706 (506, 896)	549 (442, 615)
Vitamin C intake (mg/d)	183 (149, 224)	177 (152, 197)	151 (135, 167)	184 (156, 221)	166 (146, 202)	151 (135, 167)
Vitamin B12 intake (μg/d)	3.9 (2.8, 5.6)	3.9 (3.1, 4.9)	2.6 (2.2, 3.2)	4.4 (3.4, 5.6)	3.8 (2.6, 4.8)	2.9 (2.3, 3.2)
Vitamin E intake (mg/d)	4.5 (3.6, 5.7)	4.4 (3.7, 5.0)	3.6 (3.2, 3.9)	4.6 (4.0, 5.6)	4.2 (3.5, 5.0)	3.7 (3.3, 4.0)
**Perinatal folic acid supplementation**						
0–4 weeks of gestation, n (%)	30 (44.8)	28 (41.8)	9 (13.4)	29 (50.0)	23 (39.7)	6 (10.3)
5–20 weeks of gestation, n (%)	35 (28.7)	40 (32.8)	47 (38.5)	35 (31.0)	28 (24.8)	50 (44.2)
>20 weeks of gestation, n (%)	4 (18.2)	5 (22.7)	13 (59.1)	4 (17.4)	9 (39.1)	10 (43.5)

^†^ Fruit, vegetable and vitamin intake data were adjusted for total caloric intake and Body Mass Index before pregnancy using quantile regression models. Kruskal–Wallis test and Pearson’s χ^2^ test; all differences between tertiles were statistically significant (*p* < 0.05). *p*, percentile; RE retinol equivalents.

## Data Availability

The dataset of the raw data analyzed in this study is available in Supplementary File 1. It is disposable for other research studies under proper reference.

## Supplementary Materials

The following supporting information can be downloaded at: https://www.mdpi.com/article/10.3390/nu14040746/s1, Supplementary File 1: Mexico Children's Hospital Food Consumption Frequency Questionnaire.
