# Supplementary material for: Diet and Maternal Obesity Are Associated with Increased Oxidative Stress in Newborns: A Cross-Sectional Study
Source: Nutrients. 2022 Feb 10;14(4):746. doi: 10.3390/nu14040746 (PMC8880599; doi:10.3390/nu14040746)
Supplement: Supplementary file 1 [file nutrients-14-00746-s001.zip › nutrients-1534686-supplementary.pdf]

HOSPITAL INFANTIL DE MEXICO FG

CUESTIONARIO DE FRECUENCIA DE CONSUMO DE ALIMENTOS

LECHE Y DERIVADOS

Trate de recordar su alimentación antes de que iniciara su embarazo y con que frecuencia solía consumir los siguientes alimentos

ALIMENTO **LACTEOS**	FRECUENCIA DE CONSUMO										Tipo de Cocción	**NO LLENAR**
	NUNCA	MENOS DE UNA VEZ AL MES	VECES AL MES	VECES A LA SEMANA			VECES AL DIA					
	(01)	(02)	1-3 (03)	1 (04)	2-4 (05)	5-6 (06)	1 (07)	2-3 (08)	4-5 (09)	6 + (10)	1 Crudo<br>2 Hervido o Cocido<br>3 Al Vapor<br>4 Horno o Asado<br>5 Microondas<br>6 Frito<br>8 No sabe	
1. Un vaso ce Leche entera											NA	I__I__I07I
2. Una cucharada de Queso fresco											NA	I__I__I07I
3. Una rebanada de Queso Oaxaca											NA	I__I__I07I
4. Una rebanada de Queso Manchego											NA	I__I__I07I
5. Una cucharada de Crema											NA	I__I__I07I
6. Un barquillo de Helado de leche											NA	I__I__I07I
7. Una taza de Yogurt											NA	I__I__I07I
8. Una rebanada de Queso fresco											NA	I__I__I07I
9. Una untada de Mantequilla											NA	I__I__I07I
10. Una untada de Margarina											NA	I__I__I07I

FRUTAS

ALIMENTO **FRUTAS**	FRECUENCIAS DE CONSUMO										Tipo de Cocción	**NO LLENAR**
	NUNCA	MENOS DE UNA VEZ AL MES	VECES AL MES	VECES A LA SEMANA			VECES AL DIA					
	(01)	(02)	1-3 (03)	1 (04)	2-4 (05)	5-6 (06)	1 (07)	2-3 (08)	4-5 (09)	6+ (10)	1 Crudo<br>2 Hervido o Cocido<br>3 Al Vapor<br>4 Horno o Asado<br>5 Microondas<br>6 Frito<br>8 No sabe	
11. Un Plátano												I__I__I__I
12. Media taza de Ciruelas<br>Con cáscara___(1)<br>Sin cáscara ____(2)												I__I__I__I<br>I__I__I
13. Un Durazno												I__I__I__I
14. Una Manzana fresca<br>Con cáscara___(1)<br>Sin cáscara ____(2)												I__I__I__I<br>I__I__I
15. Una Naranja											NA	I__I__I07I
16. Un vaso de Jugo de naranja											NA	I__I__I07I
17. Media Taza de Uvas<br>Con cáscara___(1)<br>Sin cáscara ____(2)											NA	I__I__I07I<br>I__I__I
18. Media Taza de Zarzamoras												I__I__I__I
19. Media taza de Fresas												I__I__I__I
20. Una rebanada de Melón											NA	I__I__I07I
21. Una rebanada de Sandia											NA	I__I__I07I
22. Un Mango											NA	I__I__I07I
23. Una Mandarina												I__I__I__I
24. Una Pera<br>Con cáscara___(1)<br>Sin cáscara ____(2)												I__I__I__I<br>I__I__I
25. Una rebanada de Mamey											NA	I__I__I07I
26. Una Tuna											NA	I__I__I07I
27. Un Zapote											NA	I__I__I07I
28. Una rebanada de Papaya											NA	I__I__I07I
29. Una rebanada de Piña												I__I__I__I

HUEVOS, CARNES Y EMBUTIDOS

ALIMENTO **HUEVOS, CARNES Y EMBUTIDOS**	FRECUENCIAS DE CONSUMO										Tipo de Cocción	**NO LLENAR**
	NUNCA	MENOS DE UNA	VECES AL MES	VECES A LA SEMANA			VECES AL DIA					
	(01)	(02)	1-3 (03)	1 (04)	2-4 (05)	5-6 (06)	1 (07)	2-3 (08)	4-5 (09)	6+ (10)	1 Crudo<br>2 Hervido o Cocido<br>3 Al Vapor<br>4 Horno o Asado<br>5 Microondas<br>6 Frito<br>8 No sabe	
30. Huevo<br>Cuantos? I___I												I__I__I__I<br>I__I__I
31. Una pieza de Pollo<br>Con piel ____(1)<br>Sin piel _____(2)												I__I__I__I<br>I__I__I
32. Una rebanada de Tocino												I__I__I__I
33. Una Salchicha												I__I__I__I
34. Una rebanada de Jamón											NA	I__I__I07I
35. Un bistec de Hígado												I__I__I__I
36. Un Chorizo												I__I__I__I
37. Un platillo con Carne res												I__I__I__I
38. Un Platillo con Carne puerco												I__I__I__I
39. Un platillo de Cecina												I__I__I__I
40. Un platillo con Atún											NA	I__I__I07I
41. Un platillo con Sardina											NA	I__I__I07I
42. Un platillo de Pescado fresco												I__I__I__I
43. Un pedazo de Chicharrón											NA	I__I__I07I
44. Un platillo de Barbacoa												I__I__I__I
45. Media taza de Mariscos												I__I__I__I
46. Un plato de Carnitas												I__I__I__I

VERDURAS

ALIMENTO **VERDURAS**	FRECUENCIAS DE CONSUMO										Tipo de Cocción	**NO LLENAR**
	NUNCA	MENOS DE UNA VEZ AL MES	VECES AL MES	VECES A LA SEMANA			VECES AL DIA					
	(01)	(02)	1-3 (03)	1 (4)	2-4 (05)	5-6 (06)	1 (07)	2-3 (08)	4-5 (09)	6+ (10)	1 Crudo<br>2 Hervido o Cocido<br>3 Al Vapor<br>4 Horno o Asado<br>5 Microondas<br>6 Frito<br>7 Asado<br>8 No sabe	
47. Media taza de Coliflor												I__I__I__I
48. Media taza de Brócoli												I__I__I__I
49. Media taza de Verdolagas												I__I__I__I
50. Un Elote												I__I__I__I
51. Una Papa o camote<br>Con cáscara___(1)<br>Sin cáscara____(2)												I__I__I__I<br>I__I__I
52. Media taza de Zanahoria<br>Con cáscara___(1)<br>Sin cáscara____(2)												I__I__I__I<br>I__I__I
53. Media taza de Espinacas												I__I__I__I
54. Media taza de Calabacitas												I__I__I__I
55. Media taza de Chayotes												I__I__I__I
56. Una hoja de Lechuga											NA	I__I__I07I
57. Un Jitomate en salsa o guisado												I__I__I__I
58. Un Jitomate crudo o ensalada											NA	I__I__I07I
59. Media taza de Nopalitos												I__I__I__I
60. Medio Aguacate											NA	I__I__I07I
61. Media taza de Flor de calabaza												I__I__I__I
62. Un betabel												I__I__I__I
63. Una rebanada de Cebolla												I__I__I__I
64. Un diente de Ajo												I__I__I__I

LEGUMINOSAS

ALIMENTO **LEGUMINOSAS**	FRCUENCIAS DE CONSUMO										Tipo de Cocción	**NO LLENAR**
	NUNCA	MENOS DE UNA	VECES AL MES	VECES A LA SEMANA			VECES AL DIA					
	(01)	(02)	1-3<br>(03)	1<br>(4)	2-4<br>(05)	5-6<br>(06)	1<br>(07)	2-3<br>(08)	4-5<br>(09)	6 +<br>(10)	1 Crudo<br>2 Hervido o Cocido<br>3 Al Vapor<br>4 Horno o Asado<br>5 Microondas<br>6 Frito<br>8 No sabe	
65. Un plato de Frijoles												I__I__I__I
66. Media taza de Chicharos												I__I__I__I
67. Un plato de Lentejas												I__I__I__I
68. Un plato de Habas<br>verdes I___I(1)<br>secas I___I(2)												I__I__I__I<br>I__I__I

CHILES

ALIMENTO **CHILES**	FRECUENCIAS DE CONSUMO										**Tipo de Cocción**	**NO LLENAR**
	NUNCA	MENOS DE UNA	VECES AL MES	VECES A LA SEMANA			VECES AL DIA					
	(01)	(02)	1-3 (03)	1 (04)	2-4 (05)	5-6 (06)	1 (07)	2-3 (08)	4-5 (09)	6 + (10)	1 Crudo<br>2 Hervido o Cocido<br>3 Al Vapor<br>4 Horno o Asado<br>5 Microondas<br>6 Frito<br>8 No sabe	
69. Una cucharada Chile en salsa												I__I__I__I
70. Chiles en lata											NA	I__I__I07I

CEREALES

ALIMENTO **CEREALES**	FRECUENCIAS DE CONSUMO										Tipo de Cocción	**NO LLENAR**
	NUNCA	MENOS DE UNA	VECES AL MES	VECES A LA SEMANA			VECES AL DIA					
	(01)	(02)	1-3 (03)	1 (04)	2-4 (05)	5-6 (06)	1 (07)	2-3 (08)	4-5 (09)	6 + (10)	1 Crudo<br>2 Hervido o Cocido<br>3 Al Vapor<br>4 Horno o Asado<br>5 Microondas<br>6 Frito<br>8 No sabe	
71. Tortilla de maíz ¿Cuántas cada vez? I____I											NA	I__I__I07I<br>I__I__I
72. Tortilla de harina. ¿Cuántas cada vez? I____I											NA	I__I__I07I<br>I__I__I
73. Una rebanada de Pan caja (Bimbo)											NA	I__I__I07I
74. Un Bolillo											NA	I__I__I07I
75. Una pieza de Pan dulce o galleta											NA	I__I__I07I
76. Un plato de Arroz												I__I__I__I
77. Una taza de Hojuelas de maíz u otro cereal de caja											NA	I__I__I07I
78. Una rebanada de Pastel											NA	I__I__I07I
79. Un plato de Sopa de pasta												I__I__I__I
80. Una Galleta salada											NA	I__I__I07I

BEBIDAS

S

ALIMENTO **BEBIDAS**	FRECUENCIAS DE CONSUMO										Tipo de Cocción	**NO LLENAR**
	NUNCA	MENOS DE UNA VEZ AL MES	VECES AL MES	VECES A LA SEMANA			VECES AL DIA					
	(01)	(02)	1-3 (03)	1 (04)	2-4 (05)	5-6 (06)	1 (07)	2-3 (08)	4-5 (09)	6 + (10)	1 Crudo<br>2 Hervido o Cocido<br>3 Al Vapor<br>4 Horno o Asado<br>5 Microondas<br>6 Frito<br>8 No sabe	
107. Un Refresco mediano											NA	I__I__I07I
108. Una taza de Atole											NA	I__I__I07I
109. Una Taza de Café negro I___I(1) o Instantáneo I___I(2)											NA	I__I__I07I<br>I__I__I
110. Una taza de Té negro											NA	I__I__I07I
111. Una taza de Té de hierbas											NA	I__I__I07I
112. Una copa de Vino tinto											NA	I__I__I07I
113. Una copa de Vino blanco											NA	I__I__I07I
114. Una Cerveza											NA	I__I__I07I
115. Una Bebida con ron, brandy o tequila											NA	I__I__I07I

GRASAS Y ACEITES

S

ALIMENTO **GRASAS Y ACEITES**	FRECUENCIAS DE CONSUMO										Tipo de Cocción	**NO LLENAR**
	NUNCA	MENOS DE UNA VEZ AL MES	VECES AL MES	VECES A LA SEMANA			VECES AL DIA					
	(01)	(02)	1-3 (03)	1 (04)	2-4 (05)	5-6 (06)	1 (07)	2-3 (08)	4-5 (09)	6 + (10)	1 Crudo<br>2 Hervido o Cocido<br>3 Al Vapor<br>4 Horno o Asado<br>5 Microondas<br>6 Frito<br>8 No sabe	
116. Aceite vegetal											NA	I__I__I07I
117. Aceite de maíz											NA	I__I__I07I
118. Aceite de olivo											NA	I__I__I07I
119. Una cucharadita Mayonesa											NA	I__I__I07I
120. Una cucharadita Manteca											NA	I__I__I07I

PLATILLOS TIPICOS

ALIMENTO **PLATILLOS TIPICOS**	FRECUENCIAS DE CONSUMO										Tipo de Cocción	**NO LLENAR**
	NUNCA	MENOS DE UNA VEZ AL MES	VECES AL MES	VECES A LA SEMANA			VECES AL DIA				1 Crudo<br>2 Hervido o Cocido<br>3 Al Vapor<br>4 Horno o Asado<br>5 Microondas<br>6 Frito<br>8 No sabe	
	(01)	(02)	1-3 (03)	1 (04)	2-4 (05)	5-6 (06)	1 (07)	2-3 (08)	4-5 (09)	6 + (10)		
121. Tacos al pastor												I__I__I__I
122. Gorditas												I__I__I__I
123. Pozole											NA	I__I__I07I
124. Quesadilla												I__I__I__I
125. Pambazo												I__I__I__I
126. Un Tamal											NA	I__I__I07I
127. Un Sope												I__I__I__I
